# Standing orders for influenza and pneumococcal polysaccharide vaccination: Correlates identified in a national survey of U.S. Primary care physicians

**DOI:** 10.1186/1471-2296-13-22

**Published:** 2012-03-20

**Authors:** Steven M Albert, Mary Patricia Nowalk, Michael A Yonas, Richard K Zimmerman, Faruque Ahmed

**Affiliations:** 1 Department of Behavioral and Community Health Sciences, Graduate School of Public Health, University of Pittsburgh, Pittsburgh, PA, USA; 2 Department of Family Medicine and Clinical Epidemiology, School of Medicine, University of Pittsburgh, Pittsburgh, PA, USA; 3 Centers for Disease Control and Prevention, Atlanta, GA, USA

**Keywords:** Adult immunizations, Influenza vaccine, Pneumococcal pneumonia vaccine, Standing orders

## Abstract

**Background:**

Standing orders programs (SOPs) allow non-physician medical staff to assess eligibility and administer vaccines without a specific physician's order. SOPs increase vaccination rates but are underutilized.

**Method:**

In 2009, correlates of SOPs use for influenza vaccine and pneumococcal polysaccharide vaccination (PPV) were assessed in a nationally representative, stratified random sample of U.S. physicians (n = 880) in family and internal medicine who provided office immunization. The response rate was 67%. Physicians reporting no SOPs, only influenza SOPs, and joint influenza and PPV SOPs were compared using multinomial and logistic regression models to examine individual and practice-level correlates.

**Results:**

23% reported using SOPs consistently for both influenza vaccine and PPV, and 20% for influenza vaccination only, with the remainder not using SOPs. Practice-level factors that distinguished practices with joint influenza-PPV SOPs included perceived practice openness to change, strong practice teamwork, access to an electronic medical record, presence of an immunization champion in the practice, and access to nurse/physician assistant staff as opposed to medical assistants alone.

**Discussion:**

Physicians in practices with SOPs for both vaccines reported greater awareness of ACIP recommendations and/or Medicare regulations and were more likely to agree that SOPs are an effective way to boost vaccination coverage. However, implementation of both influenza and PPV SOPs was also associated with a variety of practice-level factors, including teamwork, the presence of an immunization champion, and greater availability of clinical assistants with advanced training.

**Conclusions:**

Practice-level factors are critical for the adoption of more complex SOPs, such as joint SOPs for influenza and PPV.

## Background

In the United States (U.S.) yearly adult influenza vaccination coverage remains less than optimal, with 65.6% of adults aged ≥ 65, 40.1% of adults aged 50-64, and 23% of adults aged 18-49 receiving influenza vaccine in 2009 [1]. Low vaccination coverage is evident for pneumococcal polysaccharide vaccination (PPV) as well. Among adults aged 65+, 60.6% report ever receiving PPV, and among those at high-risk aged 19-64, only 17.5% report ever receiving PPV [2]. Although self-reports of vaccination may be subject to inaccurate recall [3], it is clear that missed opportunities to vaccinate adults in primary care are common and an importa77; manuscript, 77; manuscript, 77; manuscript, 77; manuscript, nt cause of low vaccination rates. Missed opportunities for vaccination often result from constraints on physician time and attendance to patients' other priorities [4,5]. Cost or availability of vaccine is less important as a barrier to vaccination in the U.S. for two reasons: 1) key national social insurance programs, such as Medicare and Medicaid, cover both the costs of the vaccines and administration by recognized providers https://www.cms.gov/Immunizations/; and 2) the Affordable Care Act mandates coverage of vaccines by private insurers.

Less than optimal vaccination coverage is typical in Europe as well. A survey of the 27 member states of the European Union, plus Iceland and Norway, during the 2006-2007 influenza season found that influenza vaccination in people aged 65+ ranged from 32.1% in Austria to 82.1% in the Netherlands. Western European countries (except for Austria and Finland) achieved the 2006 WHO goal of at least 50% coverage in the elderly, but only the Netherlands reached the 2010 WHO target of 75% coverage [6]. While most Western European countries recommend PPV for the elderly, countries vary in age guidelines and recommendations for repeat vaccinations. A systematic review found large cross-national differences in vaccination coverage [7].

Standing orders programs (SOPs) for immunization are typically facility-based policies that enable non-physician medical personnel to assess a patient's immunization status and administer vaccines without direct physician involvement. In 2002, U.S. Medicare regulations prohibiting SOPs for medication administration were modified to exclude influenza and pneumococcal vaccinations [8].

Introduction of SOPs has been shown to increase influenza and pneumococcal vaccination rates in inpatient settings [9,10]. In outpatient settings, SOPs improved influenza vaccination rates 27% in a general elderly patient population [11] and among cardiovascular patients attending a lipid clinic [12]. In a study of patients in dialysis clinics, SOPs did not increase influenza vaccination, but significantly increased PPV and hepatitis B vaccination rates [13]. In a systematic review, combination interventions which included SOPs were also effective, raising adult immunization rates by a median of 16% [14]. A meta-analysis of interventions to increase adult immunizations found that organizational change had the highest impact, though the effect of SOPs was not explicitly assessed [15]. The U.S. Advisory Committee on Immunization Practices (ACIP) [16], the Task Force for Community Preventive Services [14], and the Southern California Evidence-Based Practice Center-RAND [17] have all endorsed SOPs to reduce missed opportunities and raise vaccination rates, yet SOPs are not commonly used in the outpatient setting. Among primary care physicians, outpatient use of SOPs was 33% prior to the change in Medicare regulations [18] and since then has increased to just 42% for influenza vaccine [19].

Research on use of SOPs for influenza vaccine and PPV outside the United States is relatively scarce. However, evidence suggests that implementation of SOPs is effective in other countries. In a Canadian antenatal setting, for example, a single nurse, tasked with simply approaching patients to offer and administer vaccine, was able to boost influenza vaccination coverage to nearly half of clinic attendees [20]. A similar approach to universal screening for vaccination in the case of hepatitis B led to marked increases in uptake in Denmark [21]. Studies suggest that in the absence of SOPs or some other concerted effort, opportunities to vaccinate the elderly are routinely missed. For example, one Australian study found that hospitalized elderly who expressed interest in influenza vaccine and PPV went unvaccinated in routine care [22]. This situation may be changing. Recommendations from public health organizations in Europe now include use of SOPs: "... The clinician must be in the habit of using every visit opportunity (for whatever reason) to check vaccination status. This means empowering administrative, reception and clerical staff to evaluate vaccination history, eligibility and contraindications through standing orders" [23].

Suboptimal adult vaccination rates and low reported use of SOPs suggest that there are barriers to SOP implementation. Primary care providers in outpatient settings vary in their acceptance and use of SOPs for influenza vaccine and PPV [13,24]. The purpose of this study was to identify factors that impede or promote consistent use of SOPs for adult vaccinations and to examine factors that distinguish practices that consistently use SOPs for both influenza vaccine and PPV, practices that use SOPs for influenza vaccine alone, and practices that have no SOPs for either vaccine.

## Methods

### Sample

Physicians selected to receive a mailed survey were sampled from the American Medical Association's (AMA) master list of outpatient-based family physicians (n = 45,000) and general internists (n = 45,000) to obtain a nationally representative sample. The sample size required to detect a 10% difference in proportions of SOP implementation between the two specialty groups with a power of .80 and alpha of *P *< 0.05 was 816 (408 per group). Assuming a 50% response rate and loss due to bad addresses, we sampled 1640 physicians (820 internists and 820 family physicians) nationwide [19]. A post hoc calculation of power based on the reported prevalence of SOPs in the sample suggests we had 90% power to identify differences between SOP adopters and non-adopters as small as 10% in factors associated with adoption of SOPs. No physician assistants or nurse practitioners were sampled.

The survey was mailed with a cover letter signed by representatives of the Centers for Disease Control and Prevention (CDC), the American Medical Association (AMA), and the principal investigator. Attached to the survey was a $5.00 cash incentive to complete the questionnaire. Respondents were asked to return the completed questionnaire in a self-addressed stamped envelope. Non-respondents received a second mailed survey approximately 8 weeks after the first. After another 8 weeks, non-respondents were telephoned to request participation or to complete the survey by phone. Physicians were excluded if they were no longer in primary care practice, did not immunize in their practice, did not treat adult patients, or did not answer questions pertaining to influenza vaccination or PPV.

### Survey instrument

The survey questionnaire (see Additional file 1) was developed using aspects of the Awareness-to-Adherence model [25], which predicts physician behavior regarding new guidelines for care, and diffusion of innovation theory, which identifies predictable patterns of program adoption [26,27]. These topics were informed by findings from focus groups and interviews, which were conducted beforehand. The three focus groups involved primary care providers, including physicians as well as nursing staff. They were conducted in local sites selected for diversity in patient populations and variation in use of SOPs for adult immunizations. Focus group discussions were facilitated by a qualitative researcher who followed a written protocol developed by the research team.

Survey items were also informed by PRECEDE-PROCEED, a systematic process to evaluate health problems and design intervention programs [28]. PRECEDE is an acronym for Predisposing, Reinforcing, and Enabling Constructs in Educational Diagnosis and Evaluation. The questionnaire consisted of 22 closed-ended questions and covered demographics; practice characteristics; awareness, agreement, and use of SOPs for adult immunizations; barriers to and facilitators of SOPs; and physician attitudes regarding SOPs. It was designed to be concise to encourage response by busy physicians. The questionnaire was pilot tested with several local primary care physicians and revised as appropriate.

### Data processing and analyses

Data from returned surveys were entered using a double-entry protocol and compared using an automated procedure to identify discordant values. The files differed in < 5% of data points and differences were reconciled in each case. The final survey data file was merged with the file provided by AMA to add physician demographic, training, and practice indicators. Missing values overall, accounted for < 5% of the data. For continuous variables, mean values for the appropriate measure were calculated. The survey and research protocol were approved by the University of Pittsburgh Institutional Review Board.

For the survey, standing orders were defined as "an office policy that allows non-physician staff to screen adults for influenza and PPV and administer either vaccine to eligible adults without getting a specific order from the patient's physician." The question used to elicit SOPs status asked physicians to choose among the following categories of SOPs use in their practice: (i) no standing orders and no interest in implementing them, (ii) no standing orders but interest in implementing them, (iii) standing orders but inconsistent use, and (iv) consistent use of standing orders. The question was asked separately for influenza immunization and PPV. Combining answers to the two questions allowed us to identify physicians who reported no consistent use of SOPs for either vaccine, consistent use of SOPs for influenza vaccine only, or consistent use of SOPs for both vaccinations. (Physicians rarely reported SOPs for PPV in the absence of SOPs for influenza vaccination; see below.) The three groups were compared in univariate analyses (*χ*^2 ^for proportions, one-way analysis of variance for continuous measures) to identify significant correlates of SOPs status.

Multinomial logistic regression models were developed to examine differences between groups. In multinomial models, the two consistent SOP groups (influenza vaccine only, influenza vaccine and PPV) were each compared to physicians reporting no or inconsistent use of SOPs. A final logistic regression model was developed to examine how physicians reporting consistent SOPs for both vaccines differed from those reporting consistent use of SOPs for influenza vaccine only. For the multivariable analyses, odds ratios and 95% confidence intervals were estimated. Interaction effects were assessed but none was significant. In developing the regression models, we included variables significant in univariate analyses (*P *< .05) but also physician age and length of time in practice because these may be correlated with knowledge of SOPs and attitudes toward their use. An additional model was developed that excluded physician age and length of time in practice as covariates. Odds ratios from the alternative model did not appreciably differ and no changes in significant predictors were identified.

The following predictors of SOPs status were investigated:

*Physician-Level Factors*. Indicators drawn from the AMA file included age, time since receiving medical degree, medical specialty (family medicine or internal medicine), and board certification. The mailed survey asked whether physicians had received their medical degrees outside the U.S. and included the following indicators of physician knowledge of SOPs: whether they were aware of ACIP recommendations or Medicare regulations and whether they agreed that SOPs are effective in boosting vaccination coverage.

*Practice-Level Factors*. Physicians reported on practice-level factors likely to influence adoption or consistent use of SOPs. These included perceptions of how open their practice is to change or innovation, whether their staff demonstrates strong teamwork, and whether physicians are primary decision makers for the practice. Additional factors included access to an electronic medical record, having an immunization champion (a clinical staff member who promotes and encourages immunization) on site, size of practices (recorded as solo, 2-4 or 5+ physicians), help available to physicians (how many helpers per provider recorded as 1 helper per 2+ providers, 1 helper per provider, or two helpers per provider); type of help (advanced (PA/CRNP), nurse-level (RN or LPN) assistance, or medical assistant), and organization of the practice (comparing practices that are part of large health plans or corporations to other types). The survey instrument can be found in the Additional file 1.

## Results

Of the 1640 physicians contacted by mail, 16 letters were returned by the post office as undeliverable, and 107 physicians were no longer in practice, no longer in primary care (e.g., hospitalists), deceased, or unknown at the practice, leaving 1517 eligible physicians. Of these, 1015 physicians returned surveys for a response rate of 67%. Participation was higher among family physicians (68.9%) than internists (64.8%; *P *< .01), and among board certified (68.9%) than non-board certified (60.7%; *P *< .01) physicians. Participants and nonparticipants did not vary by age (mean = 50.7 years), length of time in practice (mean = 23 years), domestic vs. international training, or geographic region. Among participating physicians, 81.9% received their medical degree in the U.S. and 79.5% were board certified. The modal practice pattern for physicians in the sample was an independent practice (51%) in a suburban setting (45.3%). The next most common practice setting was to be based in a large corporate health system (25.5%) in an urban setting (31.2%).

Of the 1015 physicians who completed the survey, 115 did not immunize adults in their practices, leaving 900 physicians in the sample. Of these 900, 20 did not respond to questions specific to influenza vaccination. Thus, the analytic sample consisted of 880 physicians who provided information on SOPs for influenza vaccination and PPV. Consistent use of SOPs for pneumococcal vaccination in the absence of similar use of SOPs for influenza vaccination was rare; in fact, only one respondent in the sample fell into this category. By contrast, SOPs for influenza vaccination without standing orders for PPV were common. Given this distribution, we established three SOP groups: no consistent use of SOPs for either vaccination, including the one physician reporting SOP use for PPV only (n = 502, 57.0%), consistent use of SOPs for influenza vaccination only (n = 175, 19.9%), and consistent use of SOPs for both vaccinations (n = 203, 23.1%). Forty-three percent of the sample reported SOPs for influenza vaccination (with or without concomitant SOP for PPV). Physicians in the three groups did not differ in age, length of time in practice, U.S. medical training, board certification status, or specialty.

Differences among the three SOPs groups are shown in Table 1. Across the three groups, some correlates increased uniformly as use of SOPs increased, for example, awareness of ACIP recommendations and/or Medicare regulations, belief that SOPs enhance adult vaccination rates, having an immunization champion in the practice, agreement that SOPs are effective in boosting vaccination coverage, access to an electronic medical record (EMR), and having staff who are open to innovation and who work well together. Training level and number of clinical assistants were significantly related to use of SOPs, with practices reporting a greater staff to clinician ratio and those with more highly trained assistants (PA/CRNP or RN/LPN) more often reporting consistent use of SOPs. The proportion reporting 2 helpers per provider was 69.3% in the consistent influenza/PPV SOP group vs. 56.3% in the consistent influenza only group and 46.3% in the no consistent SOP group. Pairwise differences across these groupings were significant in post hoc comparisons. Number of physicians in a practice was not a significant correlate of SOP group.

**Table 1:** Characteristics of Physicians/Practices Reporting No Consistent Use of Standing Orders Programs (SOPs), Consistent Use of SOPs for Influenza Vaccine Only, and Consistent Use of SOPs for Both Influenza Vaccine and PPV*

	No Consistent Use of SOPs (n = 502)	Consistent Use of SOPs for Influenza Vaccine Only (n = 175)	Consistent Use of SOPs for Both Influenza Vaccine and PPV (n = 203)	*P*
***Physician Indicators***				

Age	50.4 (10.1)	50.2 (9.4)	51.8 (9.9)	.17

Years since medical degree	22.5 (10.4)	22.8 (10.0)	24.2 (9.8)	.13

Family Medicine specialty, %	48.2	38.3	42.9	.059

Board certified, %	79.7	81.1	78.3	.53

Medical degree outside US, %	21.7	17.7	20.7	.79

Physician is aware of ACIP recommendations and/or Medicare regulations, %	41.4	61.1	77.8	< .001

Physician believes SOPs are an effective way to boost adult vaccinations, %	60.2	79.4	82.3	< .001

***Practice Indicators***				

Practice is open to change or innovation, %	51.6	61.1	78.3	< .001

Strong practice teamwork, %	65.7	73.7	90.6	< .001

Practice uses electronic medical record, %	45.6	56.6	59.1	.001

Practice has immunization champion on site, %	23.3	27.4	38.9	< .001

Clinical support, %				

2 helpers per provider	46.3	56.3	69.3	< .001

1 helper per provider	20.0	21.9	11.8	

1 helper per 2+ providers	33.8	21.8	18.9	

Number of physicians in practice, %				

Solo	26.3	41.4	31.9	.11

2-4	18.9	43.4	37.7	

5+	24.1	34.0	41.4	

Type of clinical support, %				

RN/LPN	31.1	29.5	47.7	< .001

Medical Assistant	66.2	69.4	48.2	

PA/CRNP	2.7	1.2	4.0	

Practice is part of large health plan or corporation, %	23.0	34.3	27.6	.013

Physicians are primary decision makers for practice, %	63.6	55.0	58.4	.14

*PPV = Pneumococcal polysaccharide vaccine

To investigate correlates of SOPs in more detail, multinomial logistic regression models were developed to examine the entire set of correlates. These models allow a view of how physicians in each of the two SOP groups differ from the group not using SOPs. Table 2 shows odds ratios and 95% confidence intervals associated with each correlate in a model that includes variables shown earlier in Table 1, adjusted for physician age and length of time in practice.

**Table 2:** Practice Characteristics Associated with Consistent Use of Standing Orders Programs (SOPs) for Influenza Vaccination Only or Influenza and PPV, Relative to No Consistent Use of SOPs in Multinomial Logistic Regression

Characteristic	Consistent Use of SOPs for Influenza Vaccine Only OR (95% CI)	Consistent Use of SOPs for Both Influenza Vaccine and PPV OR (95% CI)
Physician is aware of ACIP recommendations and/or Medicare regulations	**2.35 (1.60, 3.46)**, ***P*< .001**	**4.46 (2.91, 6.85)**, ***P*< .001**
Physician believes that SOPs are an effective way to boost adult vaccinations	**2.97 (1.89, 4.67)**, ***P*< .001**	**3.50 (2.14, 5.71)**, ***P*< .001**
Practice is open to change or innovation	1.36 (0.79, 2.10)	**2.15 (1.33, 3.47)**, ***P*= .002**
Strong practice teamwork	1.18 (0.74, 1.91)	**2.78 (1.49, 5.21)**, ***P*= .001**
Practice uses electronic medical record	1.35 (0.89, 2.03)	**1.90 (1.22, 2.96)**, ***P*= .005**
Practice has immunization champion on site	1.12 (0.72, 1.76)	**1.94 (1.27, 2.98)**, ***P*= .002**
Clinical support		
2 helpers per provider	**2.75 (1.31, 5.79)**, ***P*= .008**	**2.22 (1.09, 4.54)**, ***P*= .028**
1 helper per provider	**2.30 (1.23, 4.30)**, ***P*= .009**	1.45 (0.78, 2.67)
1 helper per 2+ providers	1.00 (ref)	1.00 (ref)
Number of physicians in practice		
Solo	0.76 (0.41, 1.38)	0.71 (0.39, 1.30)
2-4	1.02 (0.65, 1.60)	0.72 (0.45, 1.17)
≥ 5	1.00 (ref)	1.00 (ref)
Family Medicine specialty	**1.63 (1.10, 2.42)**, ***P*= .015**	1.27 (0.89, 1.91)
Nurse-level/PA assistance rather than medical assistant	0.72 (0.48, 1.09)	**1.49 (0.99, 2.24)**, ***P*= .054**
Practice is part of large health plan or corporation	1.34 (0.85, 2.13)	1.04 (0.62, 1.72)
Physicians are primary decision makers for practice	0.94 (0.61, 1.45)	1.08 (0.68, 1.70)

Model adjusted for physician age and time since medical degree. Model *x*^2 ^= 239.7, p < .0001, R^2 ^= 0.26.

In the final adjusted model, consistent use of SOPs limited to influenza vaccine was independently and significantly associated with awareness of ACIP recommendations and/or Medicare regulations (odds ratio [OR] 2.35, 95% confidence interval [CI] 1.60-3.46), agreement that SOPs are an effective way to boost vaccination coverage (OR 2.97, 95% CI 1.89-4.67), family medicine specialty (OR 1.63, 95% CI 1.10-2.42), greater amount of help available to physicians (access to 2 assistants [OR 2.75, 95% CI 1.31-5.79]; access to 1 assistant [OR 2.30, 1.23-4.30], relative to the group with only 1 assistant for 2+ providers).

Consistent use of SOPs for both influenza vaccine and PPV was associated with the same set of factors but additional factors as well. Shared correlates included awareness of ACIP recommendations and/or Medicare regulations (OR 4.46, 95% CI 2.91-6.85), agreement that SOPs are an effective way to boost vaccination coverage (OR 3.5, 95% CI 2.14-5.71), family medicine specialty (OR 1.27, 95% CI 0.89-1.91, N.S.), and a greater staff to clinician ratio (OR 2.22, 95% CI 1.09-4.54). Correlates associated only with combined influenza and PPV SOPs included a variety of practice-level factors: practice openness to change and innovation (OR 2.15, 95% CI 1.33-3.47), strong practice teamwork (OR 2.78, 95% CI 1.49-5.21), access to an electronic medical record (OR 1.90, 95% CI 1.22-2.96), presence of an immunization champion in the practice (OR 1.94, 95% 1.27-2.98), and access to nurse/physician assistant staff as opposed to medical assistants alone (OR 1.49, 95% CI, 0.99-2.24, p = .054).

The two SOP groups were directly compared in Table 3. Physicians reporting consistent use of SOPs for both immunizations were more likely to be aware of ACIP recommendations and/or Medicare regulations (OR 1.95, 95% CI 1.18-3.24). Practice-level factors distinguishing consistent use of SOPs for both vaccines included strong practice teamwork (OR 2.24, 95% CI 1.10-4.54), having an immunization champion on site (OR 1.67, 95% CI 1.01-4.54), and access to nurse/physician assistant staff as opposed to medical assistants (OR 2.21, 95% CI 1.31-3.45).

**Table 3:** Practice Characteristics Associated with Consistent Use of SOPs for Both Influenza Vaccine and PPV* Relative to Influenza Vaccine Only in Logistic Regression

Characteristic	Consistent Use of SOPs for Both Influenza Vaccine and PPV OR (95% CI)
Physician is aware of ACIP recommendations and/or Medicare regulations	**1.95 (1.18, 3.24)**, ***P*= .010**
Physician believes that SOPs are an effective way to boost adult vaccinations	1.11 (0.60, 2.06)
Practice is open to change or innovation	1.59 (0.92, 2.75)
Strong practice teamwork	**2.24 (1.10, 4.54)**, ***P*= .026**
Practice uses electronic medical record	1.30 (0.77, 2.21)
Practice has immunization champion on site	**1.67 (1.01, 4.54)**, ***P*= .046**
Clinical support	
2 helpers per provider	0.86 (0.35, 2.14)
1 helper per provider	0.66 (0.30, 1.49)
1 helper per 2+ providers	1.00 (ref)
Number of physicians in practice	
Solo	0.87 (0.42, 1.80)
2-4	0.68 (0.39, 1.19)
≥ 5	1.00 (ref)
Family medicine specialty	0.78 (0.48, 1.26)
Nurse-level/PA assistance rather than medical assistant	**2.12 (1.31, 3.45)**, ***P*= .002**
Practice is part of large health plan or corporation	0.83 (0.47, 1.46)
Physicians are primary decision makers for practice	1.31 (0.78, 2.21)

Model adjusted for physician age and time since MD. Model *Χ*^2 ^= 55.0, *P *< .0001, R^2 ^= 0.20.

*PPV = Pneumococcal Polysaccharide Vaccination

**Figure 1:**
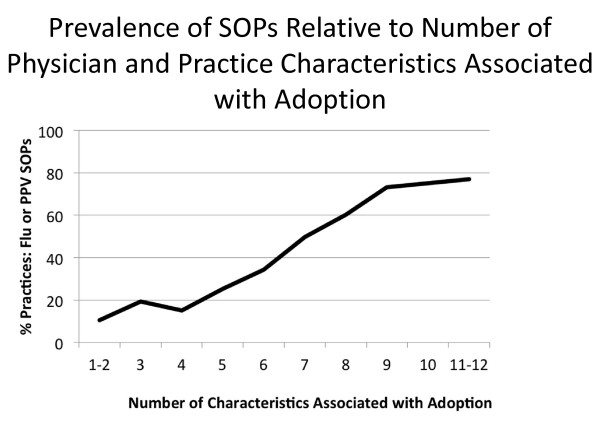
**Uploaded separately**. Prevalence of SOPs Relative to Number of Physician and Practice Characteristics Associated with Adoption.

Finally, we examined the number of physician and practice characteristics associated with adoption of SOPs and the prevalence of reported SOPs for either influenza vaccine or PPV. We summed across the 12 characteristics analyzed in the regression models and plotted adoption of SOPs against this index (Figure 1). In this sample of physicians, few respondents reported 0 or 1 characteristic (n = 3) and few reported all 12 (n = 3). Physicians reported a median of six characteristics associated with adoption. Figure 1 shows that adoption of SOPs is as low as 10.5% when only 1 or 2 factors are present and as high as 76.9% in the presence of all 11 or 12 factors.

## Discussion

In this national survey of U.S. primary care physicians treating adults, 23.1% reported using SOPs consistently for both influenza vaccine and PPV and 19.9% used SOPs only for influenza vaccination. In total, as we have reported elsewhere, 43% of physicians reported consistent use of SOPs for influenza vaccine [19]. Physicians in practices with SOPs for both vaccines reported greater awareness of ACIP recommendations and/or Medicare regulations and were more likely to agree that SOPs are an effective way to boost vaccination coverage. Implementation of both influenza and PPV SOPs was associated with a variety of practice-level factors as well, including more effective practice teamwork, the presence of an immunization champion, and greater availability of clinical assistants with more advanced training than that of medical assistants.

The CDC has recommended SOPs for adult vaccination since 2000 [16]. However, the Center for Medicare and Medicaid Services (CMS) prohibited SOPs for all medications until 2002 when CMS modified its regulations to allow SOPs for influenza vaccine and PPV [8]. It is understandable that physicians who were unaware of the change in CMS regulations might not have adopted consistent use of SOPs, as reflected in our data.

Given the myriad recommendations and policies aimed at primary preventive care, including immunizations, how can a practice keep current? One solution is for the practice to have an immunization champion, someone to interpret and communicate changes in vaccine policies and recommendations as they occur. Previous research confirms the importance of an immunization champion to improve vaccination rates. One study that included explicit training of immunization nurses to serve as clinical champions boosted influenza vaccination coverage rates in pregnant women from 2.5% to 37.4% in 2008-2009 [29]. However, it is difficult to determine the specific value of immunization champions relative to other practice-level factors, because most interventions designed to boost vaccination coverage include a variety of elements. Our findings suggest the particular value of champions for practices that have SOPs for both influenza vaccine and PPV. It would useful to determine which staff makes the most effective immunization champion and what sort of training is most appropriate. In most cases, champions are nurses but recent efforts suggest that even non-clinical staff, such as receptionists or medical records staff, can be trained as immunization champions http://www.immunizeusa.org/iz-champions/[30]. It remains unclear how such duties will be added to job descriptions or qualifications and whether additional compensation is appropriate.

Access to physician assistants or licensed nursing personnel was a significant correlate of SOPs for PPV as opposed to SOPs for influenza. Physicians in practices with access to these more highly trained personnel were twice as likely to have SOPs for PPV and influenza (Table 3). This association may be related to the complexity of PPV dosing and recommendations for individuals with high-risk medical conditions [31]. Some physicians may feel comfortable allowing clinical staff with more extensive education and training to determine eligibility for PPV and administer vaccine, as is common practice for nurses. Conversely, U.S. state laws vary regarding licensing of medical assistants [32]; these differences may result in concerns about medical assistants administering PPV under SOPs. Anecdotally, we heard reports of hesitation by medical assistants to use SOPs for PPV despite physician guidance and training using a toolkit. In contrast, influenza vaccine is administered in many locales where a physician is not even present.

The presence of an electronic medical record (EMR) was similarly associated with SOPs for PPV as opposed to SOPs for influenza. Again, this association may be a function of the complexity of PPV dosing and recommendations for individuals with high-risk medical conditions. The EMR makes it easier for practitioners to track immunization histories and to flag patients eligible for vaccination.

Teamwork is the essence of SOPs and thus the association between self-reported teamwork in the practice and use of SOPs for PPV is not surprising. Teamwork is also a key aspect of successful implementation of the patient-centered medical home and accountable care organization. Both are quality improvement models in which providing timely and appropriate preventive services, such as vaccination, is a critical element. Programs to educate staff in vaccination may help to improve perceived self-efficacy, stimulate innovation, and foster teamwork. These programs include "What works" (available at http://www2a.cdc.gov/vaccines/ed/whatworks/ce.asp and toolkits that facilitate use of SOPs http://www.immunize.org/standing-orders/ and http://www.ImmunizationEd.org/standingorders/.

We conclude that practice-level factors are critical for the adoption of more complex SOPs, such as joint SOPs for influenza and PPV. The greater clinical complexity of PPV administration may require access to electronic medical records and more highly trained auxiliary staff, for example, as well as better coordination between staff, reflected in greater perceived teamwork.

Strengths of this research include a survey that is national in scope with a high response rate for physician participants. The questionnaire was based on theoretical models designed around physician adoption of vaccines. However, surveys are subject to the limitations of self-report. While the survey covered many correlates of SOP use, it may not have captured all relevant correlates of SOP use. Also, we relied on a single physician to report on each practice and may therefore underestimate variance in delivery of vaccinations.

SOPs are underused but can have a great public health impact. For example, SOPs may help mitigate disparities in vaccination coverage in underserved populations, though we have not been able to identify research in this area. Further national efforts at clinician and medical assistant education should be considered to ensure that physicians are aware of current vaccine recommendations and policies and that all clinical staff can competently administer recommended vaccines. Practical toolkits are available to facilitate adoption of SOPs in primary care practices. Teamwork and an immunization champion can help empower clinical support staff to administer vaccines using SOPs, thus helping to reduce missed opportunities for vaccination.

## Competing interests

At the time of data analyses, Drs. Zimmerman and Nowalk had research funding and consultancy with Medimmune, Inc., and research funding from Merck, Inc., about HPV vaccine. Dr. Zimmerman also had research funding from Sanofi.

## Authors' contributions

SA performed the statistical analysis and drafted the manuscript. SA, RZ, TN, MAY, and FA conceived of the study and participated in developing the design of the research. SA and TN coordinated the survey component. All authors revised the manuscript critically for important intellectual content. All authors read and approved the final manuscript.

## Pre-publication history

The pre-publication history for this paper can be accessed here:

http://www.biomedcentral.com/1471-2296/13/22/prepub

## Supplementary Material

Additional file 1 **National Survey of Physicians about Standing Orders Programs for Adult Immunizations**. Click here for file
