# Supplementary material for: Standing orders for influenza and pneumococcal polysaccharide vaccination: Correlates identified in a national survey of U.S. Primary care physicians
Source: BMC Fam Pract. 2012 Mar 20;13:22. doi: 10.1186/1471-2296-13-22 (PMC3324392; doi:10.1186/1471-2296-13-22)
Supplement: Additional file 1 — National Survey of Physicians about Standing Orders Programs for Adult Immunizations. [file 1471-2296-13-22-S1.DOC]

Additional file 1

National Survey of Physicians about Standing Orders Programs for Adult Immunizations

1. Do you immunize adult patients in this office?

____ 00 No, *Go to Q. 9*

____ 01 Yes, *Go to Q. 2*

2. Which of the following vaccines does this office stock on site? *(Check all that apply.)*

____ 01 Influenza (in season)

____ 02 Pneumococcal polysaccharide vaccine (PPV)

3. If you do not stock the vaccine, please indicate (🗸) the reason(s) why for each vaccine.

| Barriers to stocking the vaccine in your practice | Influenza vaccine | PPV |
| --- | --- | --- |
| Insufficient profit margin |  |  |
| Little/no patient demand for the vaccine |  |  |
| Administrative burden |  |  |

4. Where are adult immunizations recorded? *(Check all that apply.)*

____ 01 Progress notes or sticker in paper chart

____ 02 Vaccine log

____ 03 Health maintenance flow sheet in paper chart

____ 04 Electronic Medical Record/Health Record

____ 05 Statewide or countywide adult immunization registry

5. Which of the following patient reminder systems are used in your office for influenza vaccine? *(Please check all that apply.)*

____ 01 Mailed reminders

____ 02 Telephone reminders

____ 03 Electronic reminders

____ 04 None of the above

6. Which of the following provider reminder systems are used in your office for adult immunizations? *(Please check all that apply.)*

____ 01 Computer generated reminders/electronic chart prompt

____ 02 Manual review of medical record and flagging at the visit

____ 03 Neither of the above

****For the purposes of this survey, standing orders refers to an office policy that allows non-physician staff to screen adults for influenza and PPV and administer either vaccine to eligible adults without getting an individual order from the patient’s physician.****

7. How would you describe your practice’s use of standing orders for adult influenza

*vaccine and PPV? (Please check (🗸) one row for each vaccine.)*

| In this practice, we are: | Influenza vaccine | PPV |
| --- | --- | --- |
| Not using standing orders, no plans to implement them |  |  |
| Not using standing orders, would like to implement them |  |  |
| Inconsistently using standing orders, some physicians use but not all |  |  |
| Consistently using standing orders |  |  |

8. With regard to initiating or maintaining a standing orders program for adult

immunization in your practice, for each of the listed adult vaccines, please indicate if

each item is: Not a barrier (0); a minor barrier (1); or a major barrier (2).

| Barriers to initiating or maintaining  a standing orders program in your practice | Influenza vaccine  0 = Not a barrier  1 = Minor barrier  2 = Major barrier | PPV  0 = Not a barrier  1 = Minor barrier  2 = Major barrier |
| --- | --- | --- |
| Insufficient patient care staff |  |  |
| Inadequate training level of patient care staff |  |  |
| Staff communication/teamwork issues |  |  |
| Lack of reliable immunization tracking system |  |  |
| Current office work flow pattern |  |  |
| Resources (time, expense) required to change office policy |  |  |
| Patient preference for physician management of vaccines |  |  |
| Physician desire for personal management of vaccinations |  |  |
| Fear of malpractice |  |  |
| Frequently changing recommendations from authorities (e.g., CDC) |  |  |
| Practice policy/physicians do not support vaccination as a preventive measure |  |  |

9. Are you aware that the CDC recommends and Medicare regulations now allow nurses and other medical support staff to administer influenza vaccine and PPV under a standing order policy, i.e., without an individual physician’s order?

____ 00 No

____ 01 Yes, aware of ACIP recommendations

____ 02 Yes, aware of Medicare regulations

____ 03 Aware of both ACIP recommendations and Medicare regulations

10. Do you agree that standing orders would be an effective way of ensuring that older

adults receive the vaccines recommended for them? Use a scale of 1 to 6 where 1

means “Strongly disagree” and 6 means “Strongly agree.” *(Circle the one number that*

*best describes your response.)*

1 . . . . . . . . . . . 2 . . . . . . . . . . . 3 . . . . . . . . . . . 4 . . . . . . . . . . . 5 . . . . . . . . . . . 6

Strongly disagree Strongly agree

11. How would you rate the level of teamwork among the staff (including providers) in

your office? Use a scale of 1 to 6 where 1 means “Poor” and 6 means “Excellent.”

*(Circle the one number that best describes your response.)*

1 . . . . . . . . . . . 2 . . . . . . . . . . . 3 . . . . . . . . . . . 4 . . . . . . . . . . . 5 . . . . . . . . . . . 6

Poor Excellent

12. How would you rate your staff (including providers) on their openness to innovation

and change? Using a scale of 1 to 6 where 1 means “Very resistant to change” and 6

means “Very open to change,” *(Circle the one number that best describes your response.)*

1 . . . . . . . . . . . 2 . . . . . . . . . . . 3 . . . . . . . . . . . 4 . . . . . . . . . . . 5 . . . . . . . . . . . 6

Very resistant to change Very open to change

13. If your practice routinely uses standing orders for adult vaccinations, which of the

following factors facilitate that process? *(Please check all that apply for each vaccine. If*

*standing orders are not used or inconsistently used, skip to Q. 14. If you do not vaccinate adults, skip to Q. 15.)*

| Facilitators of initiating or maintaining a standing orders program in your practice | Influenza vaccine | PPV |
| --- | --- | --- |
| Electronic medical record |  |  |
| Statewide or countywide immunization registry |  |  |
| Practice has a culture of openness to innovation and best practices |  |  |
| Financial incentive to vaccinate adults |  |  |
| Trust in the abilities of the support staff |  |  |
| Other, specify |  |  |

14. Does this office have an immunization champion, i.e., someone who makes special efforts to promote adult immunization?

____ 00 No

____ 01 Yes

15. Does this office have electronic medical records?

____ 00 No

____ 01 Yes

16. How much clinical help do you typically have when seeing patients? *(e.g., nurse, MA, etc.)*

____ 01 2 helpers for 1 provider

____ 02 1 helper for 1 provider

____ 03 1 helper for >2 providers

17. Who is your primary assistant?

____ 01 RN/LPN

____ 02 MA

____ 03 PA, CRNP

18. How many physicians are in this practice?

____ 01 1 (solo)

____ 02 1-5 other physicians

____ 03 >5 other physicians

19. What is the location of this practice?

____ 01 Inner-city

____ 02 Urban

____ 03 Suburban

____ 04 Rural

20. What type of practice is this?

____ 01 Independent

____ 02 Part of a small regional or other related group

____ 03 Veterans Affairs

____ 04 Part of a large corporation or health system

____ 05 Other __________________

21. What is the primary way in which policy decisions are made for this practice?

____ 01 Physician(s) decide

____ 02 Board of directors or CEO decides then informs practice

____ 03 Entire staff discusses and decides

____ 04 Committee of staff members makes recommendations and/or decisions

____ 05 Individuals make suggestions to physicians who then implement

22. Did you receive your medical degree in the US?

____ 00 No

____ 01 Yes

Thank you.
